# What is the burden of tinnitus?

**DOI:** 10.3389/fpsyg.2022.981776

**Published:** 2023-01-12

**Authors:** Helen Pryce, Nicolas Dauman, Georgina Burns-O’Connell

**Affiliations:** ^1^Department of Audiology, Aston University, Birmingham, United Kingdom; ^2^Université de Poitiers, Univ Rennes, Univ Angers, Univ Brest, RPPSY, Poitiers, France

**Keywords:** tinnitus, qualitative, burden of care theory, illness burden, treatment burden, lived experience

## Abstract

**Introduction:**

Tinnitus is a complex experience that often occurs alongside other health conditions, including hearing loss. In the UK, as in other western countries, patterns of health are changing with a rise in multi-morbidity and complexity of health conditions. As we age, we can expect to live with multiple health conditions. Burden of illness has long been recognised. Less well recognised is the burden that accumulates from the treatment of health conditions.

**Methods:**

This qualitative thematic analysis of patient accounts described the cumulative burdens of tinnitus, both the experience of hearing the tinnitus and from the treatments undertaken. Between 2017-8 we conducted interviews with 38 participants who were help-seekers in a range of contrasting UK clinical services (Physician led, Audiology led and Hearing Therapy led). We examined these interview data using reflexive thematic analysis methods to identify and explore the cumulative burdens for those who live with and seek help for their tinnitus. Specifically, we used six phased approach to determine and group themes.

**Results:**

The themes provide a coherent description of the nature of the burden that people with tinnitus experience.

**Discussion:**

In tinnitus, as with most chronic health conditions, the largest treatment workload is devolved to the patient. Patients are required to implement treatments, learn about tinnitus and find new ways of coping. Yet this work happens invisibly, without recognition from clinicians who measure outcomes but not the efforts made to achieve outcomes. Patient-centred care depends upon the recognition of the cumulative burdens that patients experience.

## Introduction - The burden of illness and burden of treatment

The ‘burden of treatment’ theory is an increasingly useful way to understand the allocation of health resources (May et al., 2014). This theory describes how health services transfer accountability and work to patients to manage long-term conditions (May et al., 2014). The management of illness involves work. This work includes coping behaviors and enduring symptoms. This work involves managing one’s own distress and negative thoughts. It involves the cognitive efforts of weighing up symptoms and bodily signs against previously held internalized illness representations. Over time as we age, we are likely to accumulate multiple health conditions all with work attached. Furthermore, health systems are increasingly moving to a position of regarding the patient as accountable for their own treatment and health. Patient capacity is regarded as a resource to be used to extend health services for example managing diabetes through self-administered insulin plus exercise, dietary adaptations etc. These then co-exist with other accumulated health conditions as we age, all of which come with their own management requirements (Spencer-Bonilla et al., 2017). In tinnitus this means that patients may be provided information to teach themselves about the nature of tinnitus (Pryce et al., 2018) and how the symptoms will interact with stress (Mazurek et al., 2015) and hearing other sounds (Pienkowski, 2019). Tinnitus is the perception of sound in the head, ear or ears when there is no external source present (Baguley et al., 2013). Psychological therapy, particularly cognitive behavioral therapy, is often cited as the most effective intervention for patients with tinnitus (Andersson, 2002; Martinez-Devesa et al., 2010; Cima et al., 2012; McKenna et al., 2017). It involves patients both attending treatment appointments and taking on responsibility to think differently about their tinnitus symptoms. It is likely that the benefit of these interventions is therefore contingent on the resources and capacity of each individual patient.

Although tinnitus is generally defined in individual terms (i.e., as the perception of sounds), qualitative research over the last decade enlightened the impact that social factors (i.e., interpersonal) had on the patients’ experience. This has been indicated by studies that incorporated the patients’ health care journey into the understanding of tinnitus-induced suffering. For instance, Dauman et al. (2017) and Pryce et al. (2019) pointed out the influence of the underestimation of tinnitus by health professionals in the worsening of patients’ distress. Since moderate tinnitus is ubiquitous in the general population, reassurance from professionals may unwittingly invalidate such distress associated with disabling tinnitus. Furthermore, medical information may also generate more worries in patients who did not anticipate having a condition that could not be cured (Andersson and Edvinsson, 2008). In other words, the individual perception of tinnitus can be influenced negatively by the perceived attitude from health professionals during consultations.

Relatively little is understood about the lived experience of tinnitus and no work has yet examined the work of living with tinnitus or the work of managing the interventions and help seeking activities for tinnitus. This matters because clinicians negotiate the workload they give to patients and may be unaware of the impact of the treatment burden they create. We set out to understand the nature of the burden of illness and treatment for tinnitus.

To do this we used qualitative methods derived from constant comparative approaches, including thematic analysis. The analysis of these themes was then informed by May et al.’s (2014) burden of illness and burden of treatment theory. We selectively considered themes that described aspects of the experience of either illness or treatment burden in particular we followed inductive methods to derive themes and then abductively reasoned how these themes described burden.

## Materials and methods

We gained Health Research Authority ethical approval from the South Birmingham research committee [16/WM/0142].

### Recruitment and sampling

**FIGURE 1:**
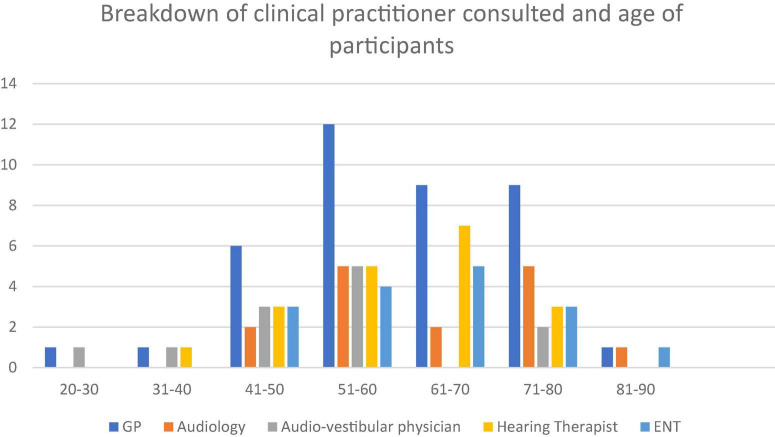
Breakdown of clinical practitioner consulted and age of participants.

**FIGURE 2:**
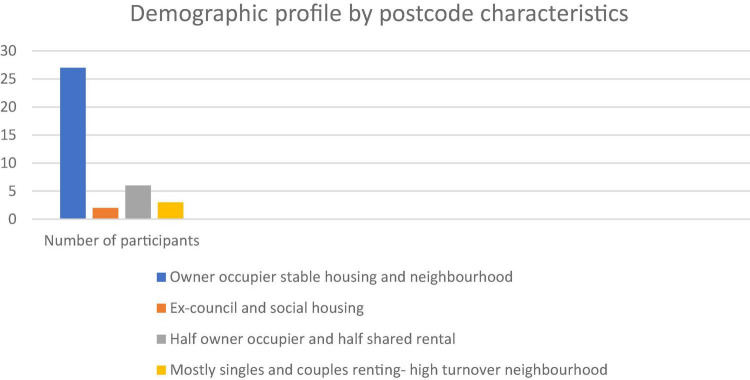
Demographic profile by postcode characteristics.

**FIGURE 3:**
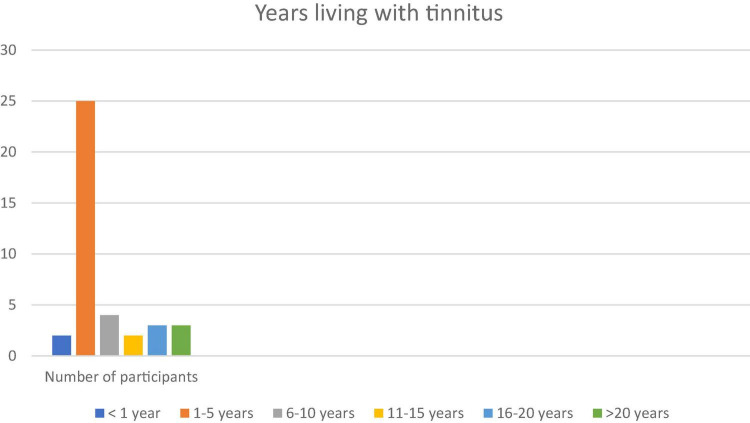
Years living with tinnitus.

Between 2017-8 we conducted 39 in depth interviews with a contrasting set of people with tinnitus. Patients were invited to participate in the research via clinical services in sites across southern England (London, Wiltshire, Bath, Somerset, and Bristol). Our sites were chosen to provide contrast in clinical experience, e.g., were Audiological Physician led, Hearing Therapist led or Audiologist led (for more details see Pryce et al., 2018). We present a relatively large sample for qualitative work in order to capture important variations in patient experience and perception of their tinnitus and healthcare experiences including consulting a range of clinicians (see Figures 1–3). Whilst the notion of data “saturation” has been widely critiqued as a neo-positivist indication of a singular “truth”; there is recognition of the importance of capturing a range of views, based on different contexts (Braun and Clarke, 2021b). Our sample included 17 females and 22 males. Our participants had been living with tinnitus from five months to 50 years. To facilitate comparison between accounts we sampled people with some contrasting features that might have different experiences of living and seeking help with tinnitus. We recruited people who were actively help seeking different sorts of hearing clinics in different parts of England with contrasting demographic features. We report on postcode demographics using Acorn profiling, the type and number of clinicians seen and the number of years living with tinnitus. The clinics involved centered provision of care from audiologists, audiological physicians or hearing therapists. Further description about the nature of the interactions between these contrasting clinicians and their patients is available (see Pryce et al., 2018). Three qualitative researchers conducted semi-structured interviews following a shared schedule of topics which explored values and preferences for care, decisions around help-seeking and day to day efforts to manage their tinnitus. These data have been previously reported on in descriptions of values and preferences in care (Pryce et al., 2018).

### Data analysis

We followed the procedures described in Braun and Clarkes reflexive thematic analysis (Braun and Clarke, 2006, 2021a). In order to maximize our understanding of the relevance of burden of treatment theory to these accounts of living with tinnitus and help-seeking, we included an additional phase to our thematic analysis and examined themes in the light of burden of care theory. This phase used abductive reasoning (Hiles, 2014) to critically examine the themes. This follows similar approaches in qualitative research (Smith and Shaw, 2017).

Firstly, two researchers (HP and GBOC) familiarized themselves with the transcribed interviews and made notes about the examples of “work” that were present. Secondly, we labeled sections of the data as illustrative of a type of work for example, managing reactions to the tinnitus, seeking information online etc. Thirdly we grouped these labeled sections into patterned themes which, illustrated different aspects of the work e.g., “illness work” (managing and responding to the tinnitus itself) and “treatment work” (managing help-seeking and the efforts required to obtain support from clinicians and enact suggested interventions). All researchers (ND, HP, and GBOC) then compared these themes and discussed the ways they operated (see Table 1 for more detail). We discussed the potential to subsume themes into overarching categories and identified their potential categories or superordinate themes that described the data set. The three superordinate themes summarized the process and attributes of work in tinnitus.

**TABLE 1:** Summary table describing the phases of data analysis.

The phase of analysis	The researchers involved	Purpose
Reading and re -reading data transcripts	HP and GBOC	Familiarization with the ways efforts to manage tinnitus are described
Notes and summary labels applied	HP and GBOC	Excerpts labeled as summary descriptors of the efforts made
Description of patterned themes that were noticed across the dataset	HP and GBOC	Labeled sections were combined to compare under broader thematic headings
Discussion about labeling	HP, GBOC, and ND	Analysis and checking of interpretations. Boundaries of themes checked. Checking inductive interpretations
Synthesis of themes into sub groups	HP, GBOC, and ND	Themes condensed into groups with common features. Inductive thematic categories agreed
Comparison of themes to burden of care theory descriptions of illness work and treatment work	HP, GBOC, and ND	Abductive comparison of themes against theory and labeling of burdens and resources

## Results

According to the study participants, the illness burden of tinnitus is twofold. On the one hand, they had to cope with the interference of tinnitus in their daily life, and adjust their behavior so they could minimize the worsening of intrusiveness. On the other hand, participants had to make sense of changes in self-perception following the onset of tinnitus, and this required additional work in order to digest the information surrounding tinnitus, i.e., that which was provided by health professionals and the information that they found on their own. More importantly, participants had to translate such information into a personal and effective routine in dealing with the symptom.

Hearing tinnitus was a constant reminder of the efforts individuals needed to make and was mentioned in each of the 39 accounts we analyzed. The work of living with tinnitus was described through coping behaviors, thoughts, efforts to switch attention, etc. The work of managing treatment described the efforts to seek help and find support and then to make sense of the interventions prescribed.

In the illness work that tinnitus required, we identified an overarching theme that is the overcoming of negative self-talk about having tinnitus. An essential feature of this basic pattern of behavior is that self-talk about tinnitus is fueled both by self-judgment on one’s behavior (e.g., self-blame, frustration or anger) and by others’ discourses about tinnitus, in particular that from the health professionals that participants met following the onset of their symptom. In most instances the GP provided professional feedback on tinnitus to the participants. This usually led patients to further medical investigations of their tinnitus symptoms, accompanied with more questions, worries and expectations about their health.

Meanwhile, no definite answer could be provided to them about the course of their tinnitus over time and how they will be able to deal with it on a daily basis. The participants had to discover it on their own, as well as the strategies that would work for them–among those that might have worked for others.

In this section, our overarching theme will be presented in relation to three main subordinate themes that allowed us to pattern out the study participants’ accounts. Negative self-talk was first reflected into the uncertainty that characterizes the experience of tinnitus as an unexpected and persistent sound in self-perception. Uncertainty was also associated with the participants’ attempts to gather multiple, often inconsistent, information surrounding the presence of tinnitus. Negative self-talk was also reflected in the experience of abandonment that was associated with the participants’ difficulties in accounting for their invisible suffering to others, in particular to health professionals. Abandonment characterized the treatment work of tinnitus as most of the work in dealing with tinnitus inevitably rested on their shoulders, a situation that would persist for an indefinite period of time. Eventually, the overcoming of negative self-talk about tinnitus implied the restoration of a sense of agency in the participants, when they could cultivate self-confidence in utilizing strategies they discovered in their own experience of tinnitus. Thus, agency was associated with the capacity to silence negative self-talk.

Each of these three patterns related to negative self-talk–uncertainty, abandonment and sense of agency– will be presented in turn and illustrated with excerpts from a diversity of participants.

### Theme 1. Uncertainty arises from the process of help-seeking for tinnitus

According to our data, negative self-talk about having tinnitus is a complex process that takes place within the interaction between patients and their clinicians. It emerges from the patients’ need for clear-cut answers about their symptoms, and medical intervention on it, that cannot be met by health professionals. Instead, the trial and error in help-seeking and in trying different interventions results in more uncertainty in patients who do not know how to get rid of tinnitus in their particular case. Thereby, negative self-talk about having tinnitus is fueled by patients’ waiting for medical answers to a daily problem they don’t know how to handle on their own. The more uncertain they feel about this handling, the more persistent would be their self-talk about lack of medical answers to their problem.

Initially, the onset of tinnitus happens as a subjective experience in one’s body. However, participants could associate the meaning of this experience not only with their own perception of it, but also with the words they heard from health professionals during the consultations. The following excerpt illustrates how those words could have iatrogenic effects, as they instilled in some individuals even more distress to the perception of the sound itself:

“I was aware of this noise in my left ear, this humming, buzzing kind of sound and I did panic a little bit. I went down to the NHS walk-in center and was told yes, that’s probably tinnitus, and I maybe panicked a bit more” (Participant 14).

Participants soon realized that there was no straightforward cure that would help them get rid of their tinnitus. Instead, they were told that further investigations would be required in order to discard underlying conditions that could be a matter of medical attention. This also contributed to the uncertainty participants were thrown into, that associated the presence of tinnitus with unexpected questions on their health status. The work of treatment in part involved understanding medical procedures and language. Participants had to interpret common phrases and the potential underlying meanings about the level of risk or harm involved, as this individual remembered:

‘The GP would send me to a consultant because what they wanted to do was to explain that there could have been some fluid or something in the ear. It could be dangerous. He said “but looking at you I don’t think there’s anything to worry about. I’m 99% sure. I’ll send you for a scan, an ear scan.” So they sent me for a scan and it came back ok of course. Then I went and saw [name of Hearing Therapist]” (Participant 32).

Having to wait for further information about their health, some participants recalled somewhat vague statements on their tinnitus over time. This also contributed to leave them in confusion and drove them to seek advice from more knowledgeable clinicians:

“I was told “Yeah, it might go away on its own, he goes it might go back to where it came from.” I thought it seemed unlikely but I sort of hung on that as a bit of hope. But then, a few weeks later it was still like it, so I went back again, saw a different doctor, and she said to me, “Have you got private health insurance at work?” And I said, “Yes, I have.” She said, “Okay, well, I’ll now refer you to an ear, nose and throat specialist” (Participant 36).

The process of clinical help-seeking could also be complicated with multiple professionals to see, in different locations, at different times. In such situations, more uncertainty may arise about potential links between tinnitus and those conditions, that would require medical interventions if needed:

”And as I say, apart from the tinnitus – which they said that wasn’t anything to do with, on top of which I just can’t be doing with sitting and waiting for scans for things that I don’t… If I’ve got pain or headaches. And we talked it all through, and then she did go away and consult with another colleague, and she said, “No, there’s really not need.” I think they were just trying to look after me basically. And I still don’t feel any particular reason to do that, I try and fit in physio for my arthritis as well, so… I work part-time as well, so I’ve still got to fit all that in” (Participant 27).

Not only professional discourses on tinnitus may have instilled uncertainty in the participants, they also could place them into a rather paradoxical situation. Indeed, participants had to rely on professionals’ views on their tinnitus and, at once, they were being told they will have to take charge of the main work in dealing with their tinnitus. How to proceed with such mysterious handling, many participants could not learn it from the health professionals they met:

“My GP, and he said “Well, yes, that’s tinnitus.” And he came out with this statement about I can’t give you a tablet and make that go away. You’ll just have to get used to it” (Participant 35).

As a consequence, filtering information and deciding on strategies was often left to the individual to put into practice. The trial-and-error approach to different therapeutic options created a burden for participants with resources (time, internet access, etc.) being used to evaluate whether a therapy might be worth pursuing. Accordingly, the uncertainty met in searching for more information about tinnitus could easily lead participants to experiencing a lack of perspective on how to handle the problem by themselves:

“So, I haven’t been very proactive in seeking it out, I did have a look online, as probably everyone does when they first get tinnitus, seeing if there’s anything out there that might help, but everything I read seemed to be very mixed, like people talked about hypnotherapy, or acupuncture but there was very few people who said, “Oh, yeah, I did that and that absolutely worked for me.” Some people said, “Yeah, I think it helped a bit, but I’m not sure”, and others say, “Oh, no it didn’t help at all.” There were some that convinced me enough for it worth taking a risk on” (Participant 28).

The lack of clear-cut information on the therapeutic outcomes was another source of uncertainty in participants. This could not provide them with a roadmap to follow, which would have motivated them to get involved in any particular treatment. As a consequence, some participants could report feelings of being left to themselves with a puzzling symptom:

“You read things on the internet and you hear people’s opinions, like, “I’ve been suffering with this,” it’s just like lots of scattered information” (Participant 33).

### Theme 2. Dealing with tinnitus on their own results in a sense of abandonment in patients

Apart from medical consultations and getting support from patient associations, most of the time participants did not have much conversation about their tinnitus with others. They had to deal with it on their own, a situation that also fueled negative self-talk about helplessness to handle the condition. Where clinicians were helpful there was still a sense of being left to make the key day to day decisions over strategies on their own. As decisions about managing the tinnitus happened the absence of clinical support, feelings of loneliness in coping with the symptom were pervasive among the participants.

Notably, such feelings were exacerbated when social interactions were non-existent, that is, during moments usually dedicated to relaxation apart from others. This is testified by these two participants who reported more difficulties to find rest because of their tinnitus. While tinnitus was in the forefront of their awareness during such moments, they could hardly talk to and share their annoyance to someone else:

“The worst time is in the morning when it’s very quiet and I’ve woken up at 5:00 sometimes 6:00” (Participant 1).

“Yes, like it’s delaying the sleeping. It’s not so easy to get off. I do eventually most nights but yes” (Participant 20).

Once participants had accessed care there was still work in terms of managing the relationship and getting the validations and support needed. Here, the sense of abandonment was associated with the subjective nature of tinnitus, especially in consultations with health professionals who could be lacking empathy with the sufferer. The experience of being left to oneself was exacerbated by individual expectations for validation of the suffering, and for medical support to cope with the distress. In the following excerpt, one can see how much the subjective nature of tinnitus leads to additional work for the patient who must convince others about the credibility of their distress. Only when distress was visible did this participant gain emotional support:

“I actually started to cry, trying to explain how it gets. And he said, “I just didn’t even realize that it felt like that for you, and I’m really sorry” (Participant 27).

Additional work for participants came from the lack of real understanding of what it is to live with tinnitus. Those who don’t have it–including some clinicians–may find it hard to grasp such a ubiquitous experience. This put an extra burden on the patient’s shoulders who asked for help. Participants described having to form relationships with clinicians, repeat aspects of their history and share information they have found on tinnitus along the way, a situation that was also required to gain knowledge and skills about how to participate in the consultation. This treatment work is summed up by this participant as follow:

“So it’s partly about educating clinicians” (Participant 38).

Another source of abandonment was the kind of advice some participants received from health professionals. Instead of tailored information that was reflective of their own situation, participants received general advice. The following participant recalled such advice about managing tinnitus:

“It was mostly just try it like this, quiet music, get yourself relaxed, just the normal sleep advice that you tend to see, get into a routine, go to bed at roughly at the same time, get up at roughly the same time, relax yourself beforehand and watch TV and stuff. I do some of that, not all of it but I’m getting better now” (Participant 36).

Ultimately, participants who received no particular answer to their worries about tinnitus were driven to explore which routines would be most helpful for them. In that regard, the experience of abandonment in dealing with tinnitus was also accompanied with the recognition that no one-fit-all solution was to be searched for. Instead, such a realization could emerge as a relief to participants who remembered their own gathering of information about tinnitus, following the consultation of health professionals:

“they gave me some forms to read and leaflets about tinnitus. I read them and I researched online and it’s true, I couldn’t find anybody who can treat it. No one said that they have any treatment for it and it just says how to live with it – how to manage it, but not treat it” (Participant 32).

At some point of the help-seeking process, many participants started to explore how available resources might fit to their particular case–rather than searching for a definite routine or treatment to adopt, without getting involved individually. Readings were presented as such valuable resources that some participants were willing to digest and put into practice:

“And she gave me lots of literature. I don’t know where I’ve put it. Lots and lots of things of dos and don’ts… But there was a lot of literature that she supplied me with and that I found very beneficial” (Participant 35).

A new attitude towards available resources emerged in some participants. This attitude implied making day-to-day decisions over strategies on their own. It also conveyed a sense of accountability in the absence of clinical support, which required to overcome scepticism about the usefulness of trying new experiences to deal with tinnitus–while previous tries may just have failed to provide any improvement. Instead of negative self-talk about lack of progress in tolerance, some participants could explore further experiences that helped others who also struggled with their tinnitus. Notably, making such decisions required them to stop dwelling upon their fate, and to trust about the positive effects to come from their initiative:

“And I’d been on the internet and found out a bit about the BTA [British Tinnitus Association] and I’ve looked at different things that were available and I mentioned something called white noise generators to her, because I’d heard about them, although I was a bit unconvinced about them” (Participant 33).

### Theme 3. A sense of agency mediates patients’ capacity to silence negative self-talk about having tinnitus

The devolution of care (general advice giving) and lack of answers from health professionals led participants to take on the responsibility of reframing their tinnitus themselves. Treatment work was characterized by seeking answers within their individual experience itself. When participants reported such self-exploration, they also had a greater sense of agency over tolerance. Moreover, such accounts suggest that having some perspective over the experience of tinnitus helped participants to silence negative self-talk about having the condition. Instead of dwelling on it, they could allocate more of their energy to find out which of their attitudes and behavior were most helpful in their particular case. In brief, their attention had been enlarged from tinnitus to themselves and their choices, and the activities they were willing to engage in. Notably, such an attitude change was associated with self-knowledge and self-growth as the following participant summarized it:

“You have to discover yourself, you have to discover what makes you relaxed” (Participant 4).

A striking feature of the data is the use of “I” as the protagonist, rather than “we” or “them” to indicate the clinicians. There was no sense that the participants who explored their experience were working alongside a clinician but rather that work is devolved to them as individuals. Another interesting indication of this attitude change toward tinnitus was the participants’ reference to greater scrutiny onto their individual experience. This is demonstrated by this excerpt from a participant who could take a broader perspective over her present situation, by reconsidering her initial drive to get rid of the problem once and for all:

“I thought well, let’s see how it goes, and I’ll seek further help if necessary” (Participant 22).

This self-exploration process led participants to try available resources they had heard about, and take steps with technical devices that provided them with some relief. Putting tinnitus in the background of their awareness allowed them to restore a sense of control in carrying on their usual business and returning to a more normal way of life:

“I bought this radio alarm clock and it had different sounds on it, sounds of nature, like waves lapping on a beach, I used to put that on at night” (Participant 35).

Having some control over self-perception in the presence of tinnitus could further lead some of them to adopt broader changes in their attitude toward their lifestyle. As an example of this, the following participant associated such restoration of agency with a greater sense of accountability in his relationship with others.

“You need to be in control of your hearing, and be one step ahead of it. You need to know what to say to people that’s going to help, and have the courage to do it. So you start to change your mentality, and you’re in charge of your hearing. And you make your life better by learning about, and learning what is going to help” (Participant 30).

Instead of suffering from the lack of a cure, some participants started to get involved in choice-making about the information they were given. This treatment work could be interesting in itself, as it led participants to better understand both the phenomenon that interfered with their life (i.e., tinnitus) and how to soften it through the use of efficient means. This work implied gaining information about a completely new area of knowledge, which participants were not aware of before they got tinnitus:

“I’ve looked at different things that were available and I mentioned something called white noise generators” (Participant 32).

Greater openness to self-experience was one of the main positive effects of the attitude change that we identified as a sense of agency in the participants. This implied a qualified sense of comparison between different circumstances of their daily life, and the associated awareness of possible softening of tinnitus when they were occupied in their daily routines. Such awareness strengthened self-confidence in participants who realized they could handle the condition on their own:

“But yeah, I began to notice this and I tried to put it to the back of my mind, I sort of I didn’t want to worry myself about it” (Participant 5).

Learning from others’ experiences of tinnitus could also be of help, as it led them to keep things in perspective:

“I try focusing on other things, and learning that other people are in a worse position than myself” (Participant 22).

One striking feature of the help-seeking process was how much agency the participant experienced in their working alliance with clinicians. Hearing Therapists were noted to provide a working alliance that supported autonomy. Looking at the following testimony, one can see how much a single, dedicated clinician showing consideration for the patient outweighs a series of consultations with emphasis on technical investigations of tinnitus:

“[Name of Hearing Therapist] was very good, I have to say. She sat me down and she explained the whole tinnitus business, and she did some diagrams – which I’ve got here somewhere, which turned out to be extremely useful” (Participant 37).

Professionals taking time to listen to the patient and providing a clear perspective on the nature of tinnitus, illustrates that the therapeutic alliance is built on the mutual relationship between patients’ growing self-confidence and trust in their clinician. When such an alliance took place, participants showed real enthusiasm about their health professionals and the help they provided them with:

“The NHS, you know the audiologist, is brilliant” (Participant 26).

Among the words that were used repeatedly in participants’ accounts, one of them was especially enlightening of the process of gaining tolerance to tinnitus. This word was “acceptance” and it was the goal of coping; a discovery associated with a restored sense of agency through the exploration of self-experience. In the following participant excerpt, such a word carried with it a substantial relief to the demanding presence of tinnitus:

“But the main thing with tinnitus, I find, one word. acceptance” (Participant 24).

### Application of burden of care theory

**FIGURE 4:**
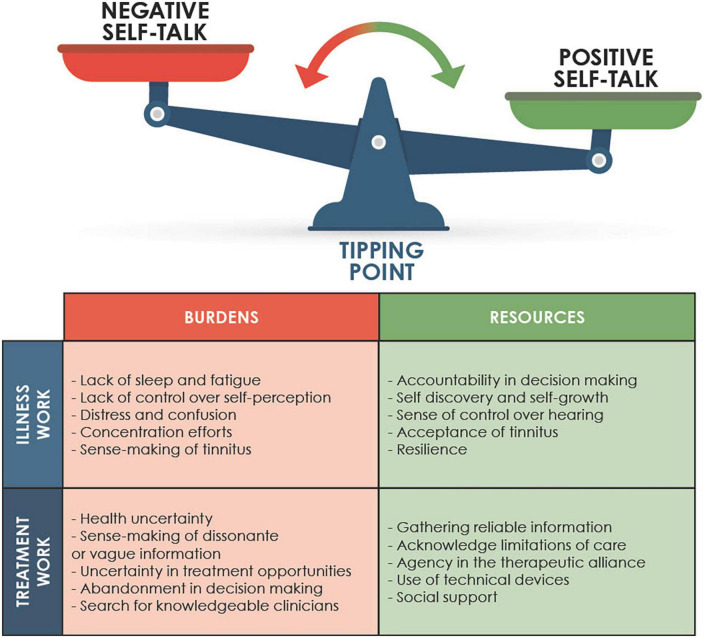
Application of burden of care theory to the thematic analysis.

The thematic analysis provided a summary of the key aspects of work incurred by tinnitus and the efforts required to mediate it. Our abductive phase involved comparing burden of care theory descriptions with the themes and categorizing them as forms of illness or treatment work. Our summary of this process is in Figure 4.

Alongside these descriptions we noted that “self-talk” described the work in the sense of self directed effort to manage internal responses to the tinnitus. Self-talk could be negative, e.g., doubts, worries or ruminations about tinnitus or tinnitus treatment; or positive, e.g., noticing one’s ability to control responses or change interpretations of the tinnitus. This process compares to May et al.’s (2014) description of “sense-making” as a part of the work of interpreting health conditions. In this case it was the means through which sense-making occurred.

## Discussion

This paper describes the characteristics of the burden of illness and burden of treatment experienced by a sample of people living with tinnitus. Burden of treatment theory proposes that variations in responses to health conditions can be understood by considering the individual resources that people have to meet the burden of living with a health condition and seeking and adopting treatment options (May et al., 2014). Our study highlights the importance of individual agency. This resonates with known influences on health behavior such as self-efficacy (Bandura et al., 1999) or internalized health locus of control (Norman et al., 1998). Burden of treatment theory proposes an extension to these models of interpersonal characteristic variation and highlights that the cognitive efforts to maintain control over a health condition (in this case, tinnitus) are a form of work. In our analysis the efforts made in self-talk comprise the clearest illustration of that work with conscious cognitive reappraisals of symptoms and re -learning of the responses to tinnitus.

This balance is individual in nature but the analysis of our data identifies a process by which the treatment work triggers some individuals to use other resources, including internal self-talk. This description is similar to the conceptualization of work required described by May et al. (2014) and referred to as ‘sense-making’. This sense-making may be influenced by peers negative self-talk. Individuals with greater resilience and increased social support may be buffered from such influence. According to burden of treatment theory, this capacity is likely to vary in important ways in individuals who are less socially privileged, have multiple health conditions with multiple treatment work demands etc. In particular, capacity varies with external resources, for example, access to information and social support. In this way the capacity for action varies depending on internal resources. The variation in tinnitus responses is documented (Cederroth et al., 2019). This may explain the heterogenous response of individuals with tinnitus and to recognize that while clinical care is helpful, it is the patient to whom the burden of self-treatment is devolved, resulting in inevitable variation. This matters when considering treatment and systemic practices. Patterns of patient preferences and values for their care have been documented elsewhere (see Pryce et al., 2018). It is likely that those managing multiple conditions, those with lower financial or social resources, those who cannot spend time and efforts learning about their tinnitus and the complexities of their treatments will be less well served the resources by services. Yet these factors are not currently explored or well understood in tinnitus research. Interventions demonstrated on average to be effective in treating tinnitus come with their own burdens. For example, attendance at a CBT group or therapy appointment take time, planning, financial resource etc. This resource may be removed with the simple change of a bus route or loss of a car or computer. It is vital that we remember the variation in capacity that people hold to engage with interventions is inherently subject to day-to-day change (May et al., 2014). The resources an individual has to reduce negative self-talk or try out a new management strategy are in competition with other demands (Erlandsson et al., 2020) and there will be variation within individuals as to the available capacity over any given day (Dauman and Dauman, 2021). It is important to recognize the demands of literacy, technical knowledge, attention and time that is inherent in these efforts. The variation in distress and coping with tinnitus is documented (Cederroth et al., 2019; Beukes et al., 2021).

The legacy health systems that were developed in the 19th Century typically separate care into specialist, fragmented services. These work well in acute care circumstances but much less well for chronic conditions (including hearing loss and tinnitus). As patients navigate these systems, the patient workload increases and, arguably, serves those most in need (those with multiple co morbid conditions) least well (Mair and May, 2014). The current moves toward “minimally disruptive medicine” are a welcome recognition of these trends (Spencer-Bonilla et al., 2017). We hope that this paper illustrates the need to broaden models to consider contextual burdens of illness and treatment in tinnitus fully.

## Data availability statement

The original contributions presented in this study are included in the article/supplementary material, further inquiries can be directed to the corresponding author.

## Ethics statement

The studies involving human participants were reviewed and approved by South Birmingham Research Committee [16/WM/0142]. The patients/participants provided their written informed consent to participate in this study.

## Author contributions

All authors contributed to data analysis, writing, and preparing the manuscript and approved the submitted version.
